# Evaluating the effectiveness of lockdowns and restrictions during SARS-CoV-2 variant waves in the Canadian province of Nova Scotia

**DOI:** 10.3389/fpubh.2023.1142602

**Published:** 2023-04-27

**Authors:** Gustavo Sganzerla Martinez, Benjamin Hewins, Jason J. LeBlanc, Pacifique Ndishimye, Ali Toloue Ostadgavahi, David J. Kelvin

**Affiliations:** ^1^Department of Microbiology and Immunology, Faculty of Medicine, Canadian Center for Vaccinology, Dalhousie University, Halifax, NS, Canada; ^2^Laboratory of Immunity, Shantou University Medical College, Shantou, Guangdong, China; ^3^Department of Pediatrics, Izaak Walton Killan (IWK) Health Center, Canandian Center for Vaccinology, Halifax, NS, Canada; ^4^Department of Pathology, Faculty of Medicine, Dalhousie University, Halifax, NS, Canada; ^5^Division of Infectious Diseases, Department of Medicine, Dalhousie University, Halifax, NS, Canada; ^6^Division of Microbiology, Department of Pathology and Laboratory Medicine, Nova Scotia Health, Halifax, NS, Canada

**Keywords:** mobility data, surveillance, infectious diseases, lockdown, COVID-19

## Abstract

**Introduction:**

After the initial onset of the SARS-CoV-2 pandemic, the government of Canada and provincial health authorities imposed restrictive policies to limit virus transmission and mitigate disease burden. In this study, the pandemic implications in the Canadian province of Nova Scotia (NS) were evaluated as a function of the movement of people and governmental restrictions during successive SARS-CoV-2 variant waves (i.e., Alpha through Omicron).

**Methods:**

Publicly available data obtained from community mobility reports (Google), the Bank of Canada Stringency Index, the “COVID-19 Tracker” service, including cases, hospitalizations, deaths, and vaccines, population mobility trends, and governmental response data were used to relate the effectiveness of policies in controlling movement and containing multiple waves of SARS-CoV-2.

**Results:**

Our results indicate that the SARS-CoV-2 pandemic inflicted low burden in NS in the initial 2 years of the pandemic. In this period, we identified reduced mobility patterns in the population. We also observed a negative correlation between public transport (−0.78), workplace (−0.69), retail and recreation (−0.68) and governmental restrictions, indicating a tight governmental control of these movement patterns. During the initial 2 years, governmental restrictions were high and the movement of people low, characterizing a ‘seek-and-destroy’ approach. Following this phase, the highly transmissible Omicron (B.1.1.529) variant began circulating in NS at the end of the second year, leading to increased cases, hospitalizations, and deaths. During this Omicron period, unsustainable governmental restrictions and waning public adherence led to increased population mobility, despite increased transmissibility (26.41-fold increase) and lethality (9.62-fold increase) of the novel variant.

**Discussion:**

These findings suggest that the low initial burden caused by the SARS-CoV-2 pandemic was likely a result of enhanced restrictions to contain the movement of people and consequently, the spread of the disease. Easing public health restrictions (as measured by a decline in the BOC index) during periods of high transmissibility of circulating COVID-19 variants contributed to community spread, despite high levels of immunization in NS.

## 1. Introduction

Restrictions in population mobility have historically been an effective method used to contain the spread of diseases (1). An example of this dates to the XIV century, where quarantines were used to control the movement of people and goods from areas where the plague (i.e., *Yersinia pestis*) was prevalent (2). During the initial outbreak of the SARS-CoV-2 pandemic, China imposed “Zero-COVID” restrictions to limit the movement of people in the greater Wuhan metropolitan area. These measures included contact limiting strategies (i.e., school and workplace closures, stay at home orders, preventing gatherings, limiting visitors to institutions, among others), which limited the spread of the disease when the number of cases was starting to increase (3). Another recent example was in India, where, following the implementation of lockdowns to contain the transmission of SARS-CoV-2, millions of workers were displaced and had to return to their hometowns; although this massive exodus was reported as a contributor to increasing cases in the country (4). Moreover, the significantly reduced human activity during lockdown periods lowered the emission of pollutant gases (5, 6), suggesting that the lockdown period contributed to cleaner, less polluted air environment (7, 8). Thus, it can be concluded that the lockdown policies implemented worldwide had a significant effect on reducing population mobility, in turn functioning to mitigate SARS-CoV-2 spread (9).

**Figure 1:**
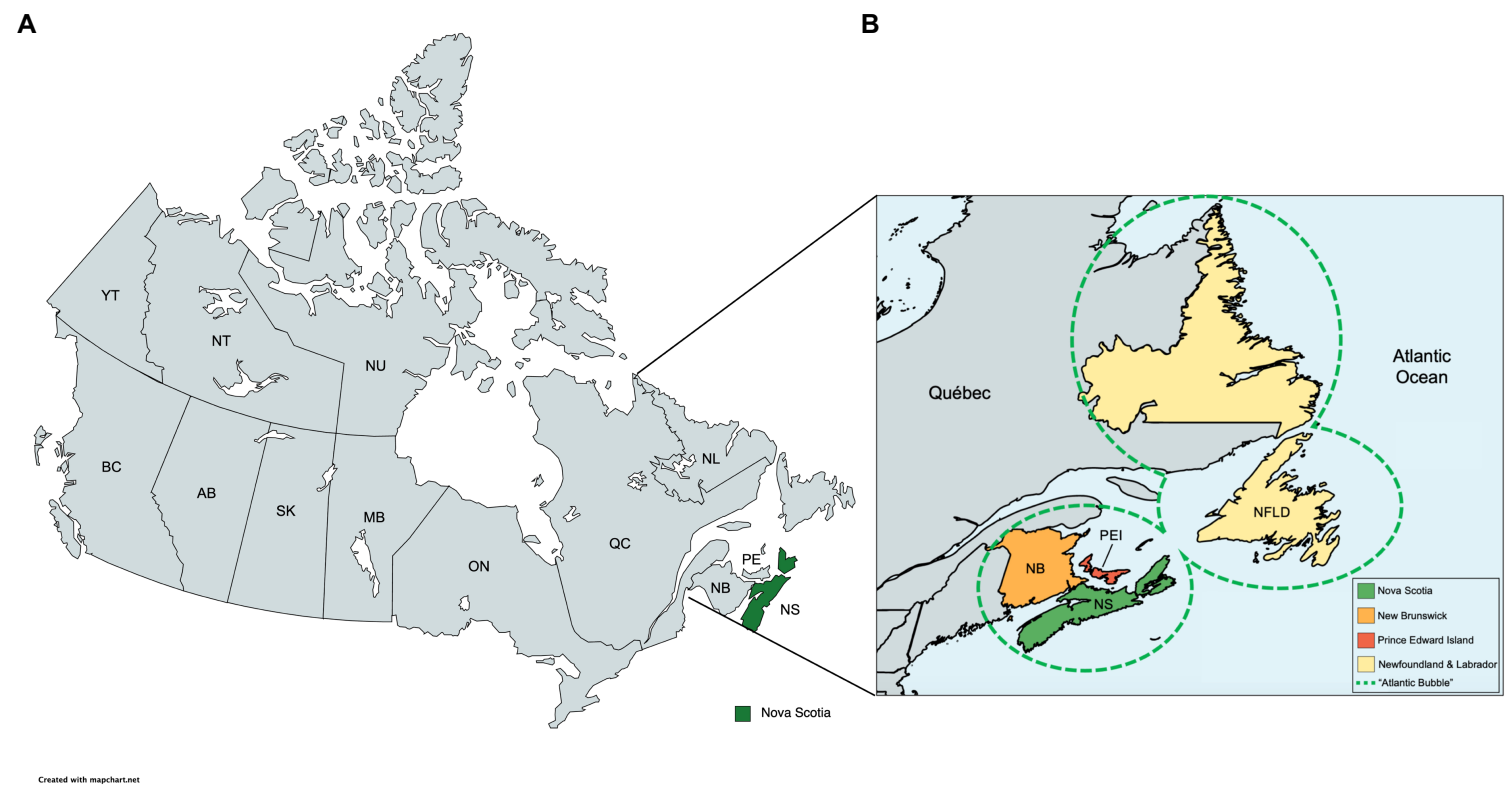
Geographical location of the Atlantic Canada. Map demonstrating the Canadian province of Nova Scotia (NS). Image created using MapChart (version 3.2.0). **(A)** Political geographical map of Canada with the province of Nova Scotia highlighted (green). **(B)** Canadian Atlantic Provinces, composed by Nova Scotia, Newfoundland and Labrador, Prince Edward Island, and New Brunswick. The dashed area represents the Atlantic Bubble.

This study focused on the Canadian province of Nova Scotia (NS) and the impact of public health measures throughout the pandemic. NS (Figure 1A) is the most populated province in Atlantic Canada, a region that also includes the provinces of New Brunswick (NB), Prince Edward Island (PE), and Newfoundland and Labrador (NL). The combined population of Atlantic Canada is around 2.5 million people over an area of 487.925 km^2^. Halifax, the capital of NS, is known as the economic hub of Atlantic Canada, containing the most frequented airport, seaport, and metropolitan area (10).

In the initial phases of the COVID-19 pandemic, the provincial government of NS created policies and enforced public health mandates that restricted the movement of individuals. These ranged from closures of schools, universities, and workplaces; cancelation of public events; restrictions on private gatherings; public transportation cancelations; stay-at-home requirements apart from individuals required for essential services; and travel restrictions (11). Public information campaigns were also used to raise awareness in the population (12). Later in the pandemic (July 2020), COVID-19-related restrictions were characterized by the installation of the “Atlantic Bubble.” The Atlantic Bubble was a pandemic mitigation effort established by the premiers of the four Atlantic provinces, which allowed unrestricted travel between the four provinces for residents of NS, NB, PE, and NL (Figure 1B), but restricted travel to the Atlantic provinces from anyone outside the bubble (except for essential services) (10).

After the initial onset of the pandemic, the multinational technology company Google used their infrastructure to collect anonymous data regarding the movement of people as a means of supporting health authorities and helping scientists understand the spatiotemporal spread of disease through changes in mobility patterns. Such data was found a valuable resource in forecasting SARS-CoV-2 transmission patterns (13) as respiratory diseases and human mobility are directly associated with routes of transmission that include contact and droplet spread (14). Additionally, indexes were created to compare the relative stringency of policy responses across jurisdictions. In Canada, the Bank of Canada (BOC) stringency index shows how the government responded to the pandemic by assessing how restrictions were implemented. It included school and university closures; workplace and office closures; public event cancelations and restrictions; restrictions on private gatherings; public transport closures; stay-at-home requirements; restrictions on intra-provincial travel; restrictions on international travel; restrictions on interprovincial travel; enforcement mechanisms for individuals; enforcement mechanisms for firms; and public information campaigns, allowing the comparison of changes in policies among provinces across time.

Throughout the initial phase of the COVID-19 pandemic in NS, both polymerase chain reaction (PCR) molecular and rapid antigen testing were readily available services (commensurate with symptoms). During the first wave (prior to Omicron), individuals experiencing symptoms consistent with COVID-19 were directed to call 811, which provides non-urgent access to health services for Nova Scotians. During an 811 call, a healthcare provider gathered information regarding symptoms (15, 16). Individuals with COVID-19 symptoms were directed to visit a provincial testing site, where a nasopharyngeal sample was taken for PCR testing. During this time, commercial reagents were scarce (and allocated federally) due to the high demand for clinical laboratories to increase testing capacity. As testing capacity increased, Canadian provinces and territories (including the Atlantic provinces) were quickly able to track new and existing cases, as well as provide sequence-based characterization of predominant circulating SARS-CoV-2 lineages. During the Omicron wave, PCR testing resources were redirected to at-risk groups, such as older adults and immunocompromised individuals. The testing and screening approach in NS was dynamic, and reflective of the specific challenges associated with each wave. Testing strategies and public health guidance evolved rapidly, based on the characteristics (i.e., transmissibility, virulence) of the circulating strain.

To further investigate the effects of restriction policies in containing the spread of SARS-CoV-2 in the Canadian province of Nova Scotia, this study aimed to evaluate the pandemic burden reflected in cases, hospitalizations, deaths, and case fatality ratio as a function of the movement of people and governmental restrictions. Accordingly, the objective (s) of this study were to determine: (1) how governmental restrictions correlate with mobility trends using publicly available mobility data; (2) whether governmental stringency was effective in controlling cases, hospitalizations, and deaths across various waves of the COVID-19 pandemic; and (3) the role vaccination played in mitigating disease burden in Nova Scotia.

## 2. Datasets and methods

### 2.1. Public COVID data

We compiled a comprehensive dataset on the COVID-19 pandemic in the province of NS, covering the period from 2020-01-25 to 2022-10-23. To obtain this data, we sourced daily figures for COVID cases, hospitalizations, vaccines, and deaths from the governmental COVID-19 Tracker Canada service (17), which provided province-wide data until 2022-07-04. We then supplemented this data with information from reliable sources such as Johns Hopkins University’s Coronavirus resources (18) and the Nova Scotia provincial pandemic figures dashboard (19). To ensure data accuracy, we processed the raw data in R (version 4.0.2) and aggregated it into weekly figures, effectively eliminating any potential backlog reporting. The resulting dataset is publicly available at https://github.com/gustavsganzerla/covid-in-ns/blob/main/pandemic_figures_NS.csv.

### 2.2. Governmental response (Bank of Canada stringency index)

At the onset of the SARS-CoV-2 pandemic, the University of Oxford’s Blavatnik School of Government developed the Oxford COVID19 Government Response Tracker (OxCGRT). This index was designed to evaluate how effectively governments worldwide were responding to the pandemic by assessing the strictness of their mandates. The score assigned to a specific country or province ranges from 0 to 100, based on a series of questions. The Bank of Canada developed a Canadian version of the score, known as the Bank of Canada Stringency Index (12). This index adapted the questions used in OxCGRT to reflect Canadian policies and practices. The index measures various indicators, including school and university closures; workplace and office closures; public event cancelations and restrictions; restrictions on private gatherings; public transport closures; stay-at-home requirements; restrictions on intra-provincial travel; restrictions on international travel; restrictions on interprovincial travel; enforcement mechanisms for individuals; enforcement mechanisms for firms; and public information campaigns. The Bank of Canada Stringency Index provides daily data for NS from 2020-02-22 to 2022-07-17. To eliminate seasonal effects, such as holidays and weekends, we processed this data into weekly variations. The resulting dataset is publicly available at https://github.com/gustavsganzerla/covid-in-ns/blob/main/pandemic_figures_NS.csv.

### 2.3. Mobility trends

We accessed community mobility reports from Google (20), which provide valuable insights into changes in the movement patterns of people residing in Nova Scotia during the pandemic period. This data was updated and available daily, spanning from 2020-02-15 to 2022-10-13. Google’s anonymized mobility records are assigned to users with their location services enabled while visiting categorized locations, such as workplaces; residential areas; public transportation; retail and recreation centers; grocery and pharmacy stores; and parks. To establish a baseline for movement, Google used a five-week period from 2020-01-03 to 2020-02-06. Any fluctuations observed in the movement patterns from this baseline value are classified as either positive or negative movement records. Our analysis involved studying the mobility data of these six categories over several weeks, allowing us to eliminate seasonal variations such as weekends and holidays. The dataset we have assembled is publicly available at https://github.com/gustavsganzerla/covid-in-ns/blob/main/mobility_NS.csv.

### 2.4. Statistical analyses and figure generation

All the figures used in this study were generated in the R statistical programming software (4.02). The packages *ggplot2* 3.3.5, *ggpmisc* 0.4.4, *tibble* 3.1.6, *egg* 0.4.5, and *zoo* 1.8–8 were used. We employed the Shapiro–Wilk test for data normality, Pearson test for correlation, Kruskal-Wallis, and ANOVA tests for mean comparison. All the statistical tests were conducted using the *stats* base R package and the *rstatix* package 0.7.0. Finally, we performed a range normalization, which places the stringency data (ranging from 0 to 100) into the range of each data with which it is compared (i.e., weekly number of cases, hospitalizations, and deaths). Equation 1 shows the normalization process:


(1) $$Z_{i}=\frac{\left(i-n_{\min}\right)}{\left(n_{\max}-n_{\min}\right)}*\left(rst_{\max}-rst_{\min}\right)+rst_{\min}\;$$

where $z_{i}$ is the ith normalized data, $n_{\min}$ is the minimum value in the stringency analysis, and $n_{\max}$ is the maximum. $rst_{\max}$ is the maximum value for cases, deaths, and hospitalizations, respectively, while $rst_{\min}$ is the minimum.

## 3. Results

### 3.1. How mobility trends in NS during the first 2 years of the pandemic are associated with governmental restrictions

**Figure 2:**
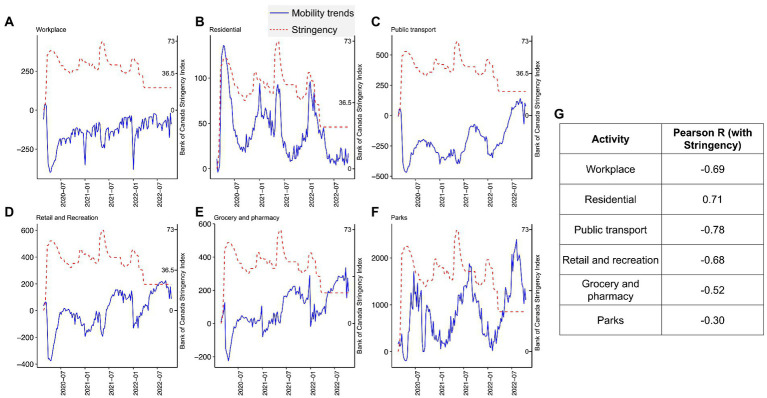
Nova Scotia mobility trends throughout the pandemic. We report mobility trends over six different activities throughout the COVID-19 pandemic. Workplace **(A)**, residential **(B)**, public transport **(C)**, retail and recreation **(D)**, grocery and pharmacy **(E)**, and parks **(F)** activities from the Google mobility reports service were considered. The 0 value in the y-axis indicates the baseline period Google assembled for the non-pandemic weeks from 3 January 2020 to 6 February 2020. In addition, we overlayed the stringency index of Bank of Canada in each one of the mobility panels. We scaled stringency with each mobility pattern following Equation 1 and replaced the variables (i.e., cases, hospitalizations, and deaths) by each one of the mobility metrics. In **(G)**, we performed a Pearson correlation analysis of the mobility trends with stringency. We report the coefficients.

To compare the relationship of changes in mobility data to changes in Nova Scotia government policy restrictions during the pandemic, we plotted the BOC stringency index values for Nova Scotia (Figures 2A–F dashed red line) with the mobility data for the following: (i) workplace; (ii) residential; (iii) public transport; (iv) retail and recreation; (v) grocery and pharmacy; and (vi) parks (Figures 2A–F, solid blue lines). After the initial onset of the pandemic, workplace (Figure 2A), public transport (Figure 2C), retail and recreation (Figure 2D), and grocery and pharmacy (Figure 2E) mobility activities dramatically decreased. Residential mobility (Figure 2B) dramatically increased, reaching the highest levels during the pandemic. We next performed a correlation analysis (Figure 2G) of mobility and the BOC stringency index (Figure 2G). We found public transport (−0.78), workplace (−0.69), and retail and recreation (−0.68) were the activities most negatively correlated to governmental restrictions as they presented negative correlation coefficients. Residential mobility (0.71) was positively correlated with the BOC stringency index.

It would appear based on the data presented in Figure 2 that the restrictive measures employed in Nova Scotia during the pandemic were effective in altering mobility patterns of the population. The strongest effects were observed in the initial stages of the pandemic. Evidence of that is the higher Pearson correlation coefficient observed between stringency and the mobility levels in the first 20 weeks of the pandemic (from 2020-02-22 to 2020-06-06), i.e., workplace = −0.89; residential = 0.85; public transport = −0.91; retail and recreation = −0.76; grocery and pharmacy = −0.56; and parks = 0.07.

**Figure 3:**
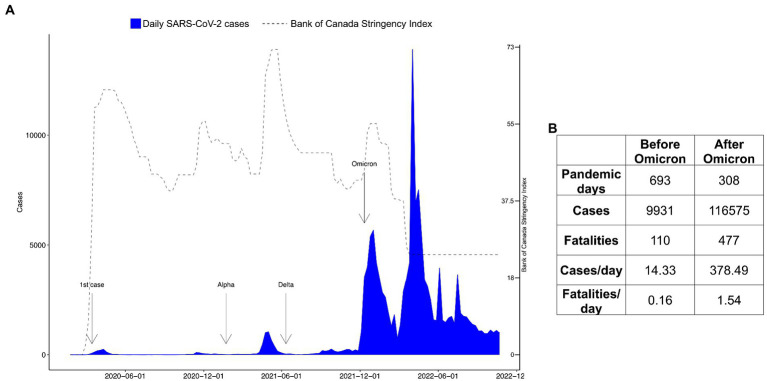
Variant timeline and pandemic figures in Nova Scotia before and after Omicron. In **(A)**, we show the weekly report on COVID-19 cases in Nova Scotia as well as a timeline of the Variants of Concern (VoCs). The first case was reported in the province on 2020-03-15. The reports of the introduction of VoCs are annotated in the plot as follows: the first case of the Beta (UK) variant on 2021-01-22; the first case of the Delta variant on 2021-06-11; and the first Omicron case on 2021-12-12. To track the VoCs, we obtained news data from https://signalhfx.ca/covid-19-in-nova-scotia-2021/ and https://ipac-canada.org/covid-19-variants-of-concern. In **(B)**, we show the number of pandemic days, number of cases, and fatalities in the time period before Omicron (from 2020-01-25 to 2021-12-12) and after Omicron (from 2021-12-13 to 2022-10-22).

The BOC stringency index fluctuated throughout the pandemic in NS (Figure 2). These fluctuations were due to restrictive policy changes made by the NS government in response to the changes in case numbers, hospitalizations, COVID-19 related fatalities, and community circulation of SARS-CoV-2, as well as other factors (4). In NS, different SARS-CoV-2 variants dominated at different times, where new variants often replaced older variants (8). Figure 3 shows the introduction of the first diagnosed cases of new variants of SARS-CoV-2, including the original Wuhan strain (D614G), SARS-CoV-2 Alpha (B.1.1.7), SARS-CoV-2 Delta (B.1.617.2), and SARS-CoV-2 Omicron (B.1.1.529). Case numbers are plotted in Figure 3 to give a perspective of the impact of SARS-CoV-2 variants on health burden in NS.

Restrictive policy measures remained in a high range (higher than 43.01, the mean value throughout the whole time series) from early 2020 to the first 2 months of 2022. Even though the restrictive policy measures were high during the first phase of Omicron, the cases dramatically increased. As Omicron was becoming the dominant strain in 2022, restrictions were reduced, as shown by the decline in the BOC stringency index (Figures 2, 3), and mobility activity returned to pre-pandemic levels for (i) workplace; (ii) residential; (iii) public transport; (iv) retail and recreation; and (v) grocery and pharmacy despite the higher number of cases, hospitalizations, and fatalities during this period.

After Omicron, the activities that were low during the initial onset of the pandemic maintained higher levels; residential mobility reached its lowest level when active cases of SARS-CoV-2 were being reported in NS. We also report a seasonal aspect in mobility trends in NS, which tends to present reduced out-of-home mobility levels (i.e., parks, retail/recreation, public transport, and grocery/pharmacy) in the winter periods (January 2021 and January 2022).

We tabulated the number of cases, hospitalizations, and fatalities during SARS-CoV-2 variants (Figure 3). The Omicron variant hit NS on 2021-12-13, and the number of cases increased dramatically in the province (Figure 3). The pre-Omicron stage encompassed 689 days (2020-01-25 to 2021-12-12) with the Omicron wave (2021-12-13 to 2022-10-23), totaling 308 days. Proportionally by the number of days, the Omicron period is 26.41-fold more transmissible and 9.62-fold more lethal than the previous waves of the virus.

### 3.2. Measuring governmental COVID-19 response

**Figure 4:**
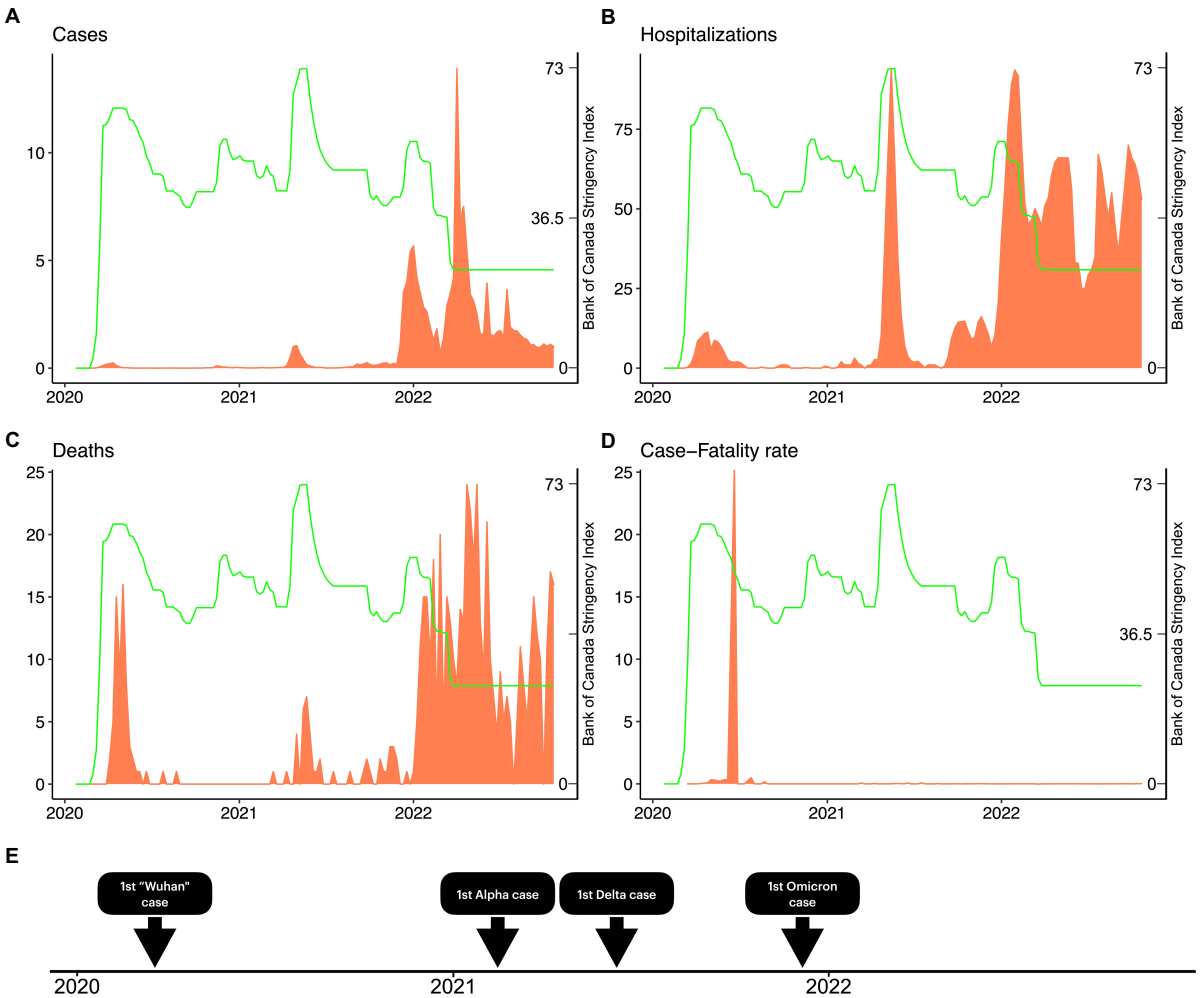
COVID-19 cases, hospitalizations, fatalities, case-fatality ratio, and the Stringency Index in Nova Scotia. We demonstrate the weekly evolution of Bank of Canada Stringency Index (green) throughout the pandemic in Nova Scotia. We also overlay the Stringency Index with the weekly number of cases **(A)**, hospitalizations **(B)**, deaths **(C)**, and case-fatality ratio **(D)**. In **(E)**, we show a timeline comprising the first case reported of the original Wuhan strain, Alpha, Delta, and Omicron strains in Nova Scotia.

We analyzed the restrictive measures implemented by NS as a response to COVID-19 through the BOC stringency index. We report the maximum stringency score (73.32) recorded from 2021-05-15 to 2021-05-22 (i.e., 2 weeks). This period accounted for 973 cases, 165 hospitalizations, 13 deaths, and case-fatality rate (CFR) 0.76%. The highest stringency recorded during Omicron was 55.50 for three consecutive weeks (from the week of 2021-12-25 to 2022-01-08). During this period, there were 14,741 cases, 115 hospitalizations, 6 deaths, and a 0.04% CFR, the later being shrunk by the cases which were 26-fold higher during Omicron. Following this period, the stringency continued to decline weekly and the minimum stringency during Omicron (i.e., 24.07) had never been matched while there were active cases of COVID-19 in NS. Moreover, we plotted the BOC stringency index with cases (Figure 4A), hospitalizations (Figure 4B), fatalities (Figure 4C), and case-fatality rate (Figure 4D). From that, we report the highest peak observed in hospitalizations also matched a high peak of stringency (2021-05-15). The results displayed here indicate a two-phased approach to the SARS-CoV-2 pandemic in NS: (i) prior to the arrival of the Omicron variant, characterized by low cases (14.33 cases/day), hospitalizations (6.28/day), deaths (0.16 death/day), higher stringency (mean 46.66 in the period), and lower mobility (mean 57.35 in the period) and (ii) after the onset of Omicron, characterized by high number of cases (378.49/day), hospitalizations (49.11/day), deaths (1.54/day), low stringency (mean 34.41 in the period), and higher mobility (mean 189.33 in the period).

### 3.3. Vaccination in Nova Scotia

**Figure 5:**
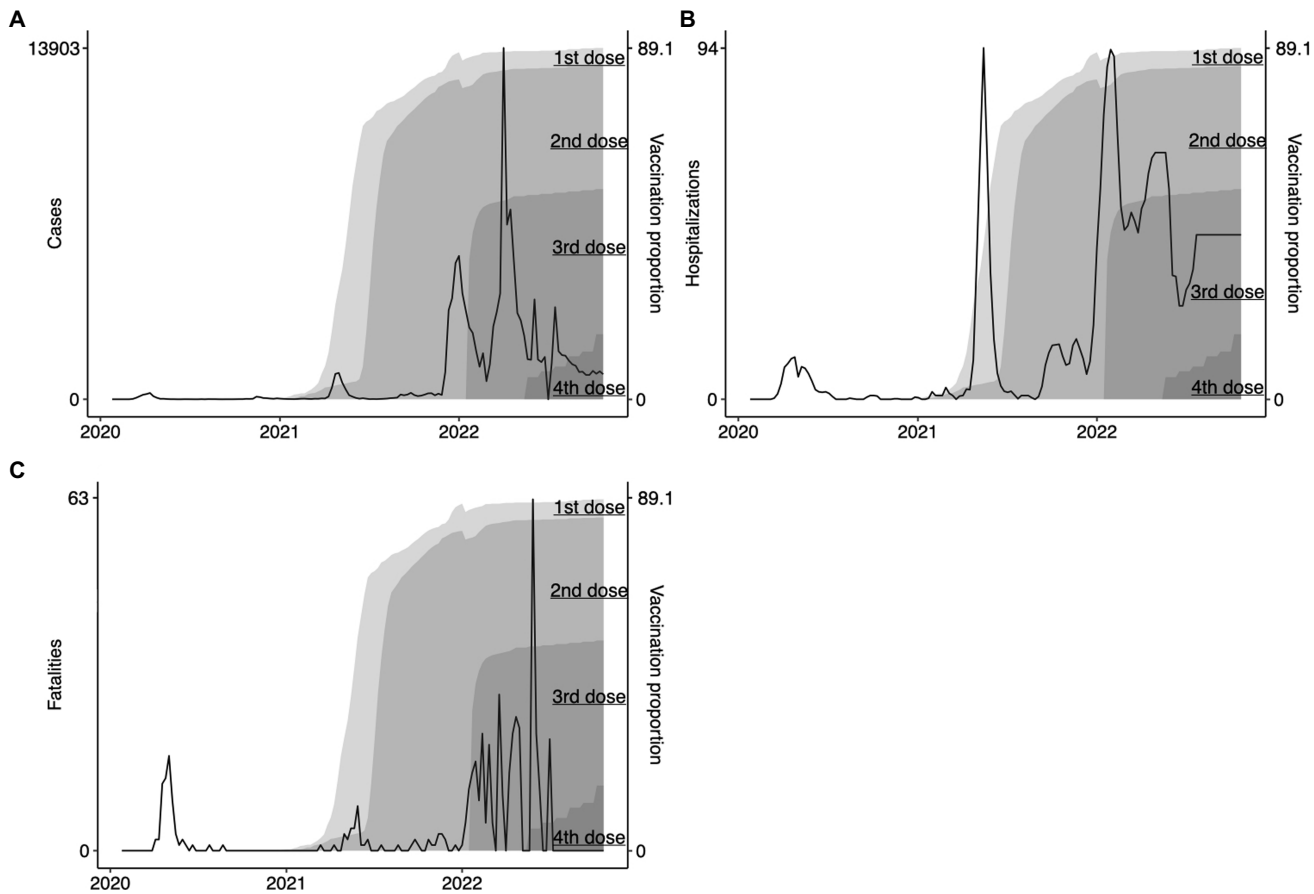
Evolution of COVID-19 vaccination in Nova Scotia. We show the evolution of vaccination rates in Nova Scotia represented in the proportion of the population that had took their 1st dose, 2nd dose, 1st booster, and 2nd booster (labeled gray areas in the right-hand side of panels **A** – cases, **B** – hospitalizations, and **C** – fatalities).

The first vaccine administered in NS took place on 2020-12-19. Up to the last collection date (2022-10-09), 89.1% of the population have taken at least one dose, 84.5% have taken two doses, while a first boost has been applied to 50.4% and a second to 16.5% (Figure 5). We also plotted how the number of cases evolved as the vaccination rate grew (Figure 5A). In the highest peak of cases reported during Omicron (i.e., 11,222 cases on the week of 2022-04-02), 88.2% took at least one dose, 83.4% took two doses, 50.4% took the first booster, and 0% had received the second booster. In Figure 5B, we show the evolution of hospitalizations and the vaccination rate. During the peak in hospitalizations (i.e., 93 hospitalizations reported in the week of 2022-01-29), 83.0% had taken at least one dose, 79.6% had received a second dose (however, the number of 1st doses is higher than 2nd doses, indicating a potential change in the number accountability), 42.3% had taken a first booster, and 0% of people had taken a second booster. Finally, during the peak of Omicron deaths on 2022-05-28 (i.e., 63 deaths recorded in a week), 88.3% of people had taken a first dose, 83.9% had taken two doses, 51.8% had taken a first booster, and 46.2% had received their second booster. Thus we suggest that the rising of Omicron pandemic figures might have been facilitated by the vaccine breakthrough caused by the Omicron variant (21).

## 4. Discussion

Our results show the SARS-CoV-2 pandemic was characterized by two distinct phases in NS. Initially, the pandemic burden was relatively low while the government implemented harsher restrictions that limited population movement. Limiting the contact of people was successful in controlling early transmission of COVID-19 (22–27). As SARS-CoV-2 evolved (culminating during the highly transmissible Omicron wave), the disease burden increased, partially, as a function of reduced government restrictions that are difficult to sustain in long-term (28, 29) and due to a general lack of adherence within the population (30). Interestingly, studies have shown that stringent, non-pharmaceutical interventions (NPIs) enforced during the COVID-19 pandemic shifted overall population mobility, but also led to instances of higher-than-average population mobility within certain regions based on population density. In Britain, for example, an increased migration of individuals was temporarily observed from high population density areas to areas of lower population density during periods of stringent NPI restrictions on mobility, suggesting mobility trended away from epicenters during the pandemic (31). This is a parameter we would like to explore further, given the increased intra-provincial travel observed during mobility restrictions and lock-down events and the increased incidence of cases contracted in Halifax, the metropolitan hub of NS. A separate mobility study in Europe further classified individual mobility based on activity, where occupation-related mobility decreased during the pandemic and ‘weekend’ activities, or activities relating to leisure (i.e., walking, jogging, weekend travel trips) increased during the same period (32). Furthermore, their data suggests that visits to parks increased during lockdown periods in Denmark, similar to findings presented in this paper. In addition to the role that behavioral tendencies and population mobility contribute to case numbers, hospitalizations, and deaths, it is crucial to consider the impact of the virulence and transmissibility of the circulating variant that co-occurs with restrictions.

The Omicron variant and its sublineages stressed the healthcare system of NS, as shown in Figure 4 by the substantial increase in cases, hospitalizations, and fatalities after the second year of the pandemic; the same pattern was observed in other countries (33). This variant can escape vaccine and infection induced immunity, resulting in a more transmissible virus (34), due (in-part) to vaccine-variant mismatch, where first, second, and third doses were designed against earlier variants such as Wuhan or Alpha (B.1.1.7) (35). Studies show that Omicron-related infections generally cause milder COVID-19 infection (36, 37), further compounded when Omicron encounters a vaccinated population (21). Infections and deaths continued to rise in NS during the Omicron wave (Figure 5), including BA.1.1.529 and sublineages XBB.1.5, BQ.1, BQ.1.1, BA.2.75, BA.4/0.5, despite widespread vaccination. This is partly attributed to breakthrough infections, which occur when those with two or more doses of a SARS-CoV-2 vaccine contract a subsequent infection (38). Omicron (BA.1.1.529) contains 30 amino acid (aa) substitutions, 6 aa deletions, and 3 aa insertions compared to ancestral strains (39, 40). These genetic and protein level mutations increase transmissibility and immune evasion of Omicron from vaccine induced and convalescent neutralizing-antibodies (nAbs). BA.4/0.5 contain additional mutations in the N-terminal domain and receptor binding domain (RBD) regions of the spike (S) (L452R, F486V, R493Q, 69-70Δ) protein not seen in BA.1.1.529. The resulting S contains a vastly modified mutational landscape compared to those of ancestral strains. Such variation causes increased vaccine-variant mismatch and consequent breakthrough infections. Evidence also suggests that BA.4/0.5 and more recent Omicron sublineages display escape from BA.1.1.529 elicited immunity in vaccinated, previously infected, and vaccinated-previously infected individuals (40). Therefore, protection offered by intramuscularly injected SARS-CoV-2 vaccines must be carefully evaluated.

Although this study provides a foundation for applying population-level mobility data as a predictive model for estimating disease burden during a pandemic, limitations in the generalizability of the results remain. For example, additional variables beyond mobility data likely play a role in transmission dynamics, such as the pathogenicity and transmissibility of the circulating SARS-CoV-2 variant, which fluctuates rapidly following genetic-and protein-level mutations in the RBD region of SARS-CoV-2. Furthermore, our model may not be generalized to account for the variable restrictive management styles of public health authorities across the globe, as the transmission dynamics of infectious diseases follow a nonlinear, non-stationary and volatile aspect (41). While some countries, regions, or provinces began to ease restrictions, other countries imposed ongoing restrictions and the level of restrictions varied following the discretion of governing health authorities. The quality and reproducibility of mobility data may also pose as a limitation, where developing countries or data restricted countries may not have equal access to this information.

The movement of a population is associated with the spreading of infectious diseases (42). In our results, Figure 2 shows that residential mobility levels were high during most of the pandemic period; however, public transport, retail/recreation, and workplace sustained low levels during the pandemic; and parks and grocery/pharmacy declined initially but then increased. It has been reported that a population that does not trust its government is less likely to adhere to restriction policies that limit their movement (43–45). Our results demonstrate an association between movement reduction and stringency within public transport, which is directly controlled by the government. Similar findings were observed in the city of Madrid, where public transport ridership decreased by 95% during the pandemic peak, compared to pre-pandemic levels (46). Therefore, we suggest that a population is more likely to adhere to restrictions imposed by a centralized government. We also demonstrate that stringency is positively, moderately correlated with residential mobility, meaning that with increasing restrictions, more people tend to stay home.

## 5. Conclusion

Our results suggest that the lower movement of people was likely one of the variables that allowed NS to maintain its low disease burden during the first 2 years of the COVID-19 pandemic. Conversely, the higher movement of people, in combination with a more transmissible viral variant, might have contributed to the increase in cases observed throughout the Omicron period. Thus, to effectively implement restrictive measures, public health should consider three important factors: (i) the nature of the circulating strain of the virus; (ii) the level of immunity within the population, including previous exposure and immunization status; and (iii) the movement dynamics of the population. The global impact of this study provides additional framework for rapidly coordinating responses to emerging SARS-CoV-2 variants and novel pandemic threats. These results will allow policymakers to accurately triage the level of risk associated with a given pandemic, dictating the appropriate restrictive measures to impose on a population to reduce pandemic burden and fatigue. This cost-effective strategy may be applied to various outbreaks, epidemics, and pandemics, functioning to inform public health agencies regarding the effectiveness of imposed restrictions and whether those restrictions are capable of containing a given infectious disease.

## Data availability statement

Publicly available datasets were analyzed in this study. This data can be found at: https://www.google.com/covid19/mobility/ (mobility reports); https://www.bankofcanada.ca/markets/market-operations-liquidity-provision/covid-19-actions-support-economy-financial-system/covid-19-stringency-index/ (Stringency index); and https://covid19tracker.ca/index.html (COVID-19 data).

## Author contributions

GSM, BH, and DJK designed the study. BH wrote the manuscript. GSM prepared the figures. JJL and PN provided meaningful public health interpretations of the results. All authors contributed to the article and approved the final submission.

## Funding

This work was supported by awards from Research Nova Scotia (DJK), the Canadian 2019 Novel Coronavirus (COVID-19) Rapid Research Funding initiative (CIHR OV2–170357), the Canadian Institutes of Health Research (CIHR), Atlantic Genome/Genome Canada (DJK), Li-Ka Shing Foundation (DJK), Dalhousie Medical Research Foundation (DJK). DJK is the Canada Research Chair in Translational Vaccinology and Inflammation.

## Conflict of interest

The authors declare that the research was conducted in the absence of any commercial or financial relationships that could be construed as a potential conflict of interest.

## Publisher’s note

All claims expressed in this article are solely those of the authors and do not necessarily represent those of their affiliated organizations, or those of the publisher, the editors and the reviewers. Any product that may be evaluated in this article, or claim that may be made by its manufacturer, is not guaranteed or endorsed by the publisher.
